# Supplementary figures and images for: A comprehensive DNA barcode inventory of Austria’s fish species
Source: PLoS One. 2022 Jun 9;17(6):e0268694. doi: 10.1371/journal.pone.0268694 (PMC9182252; doi:10.1371/journal.pone.0268694)

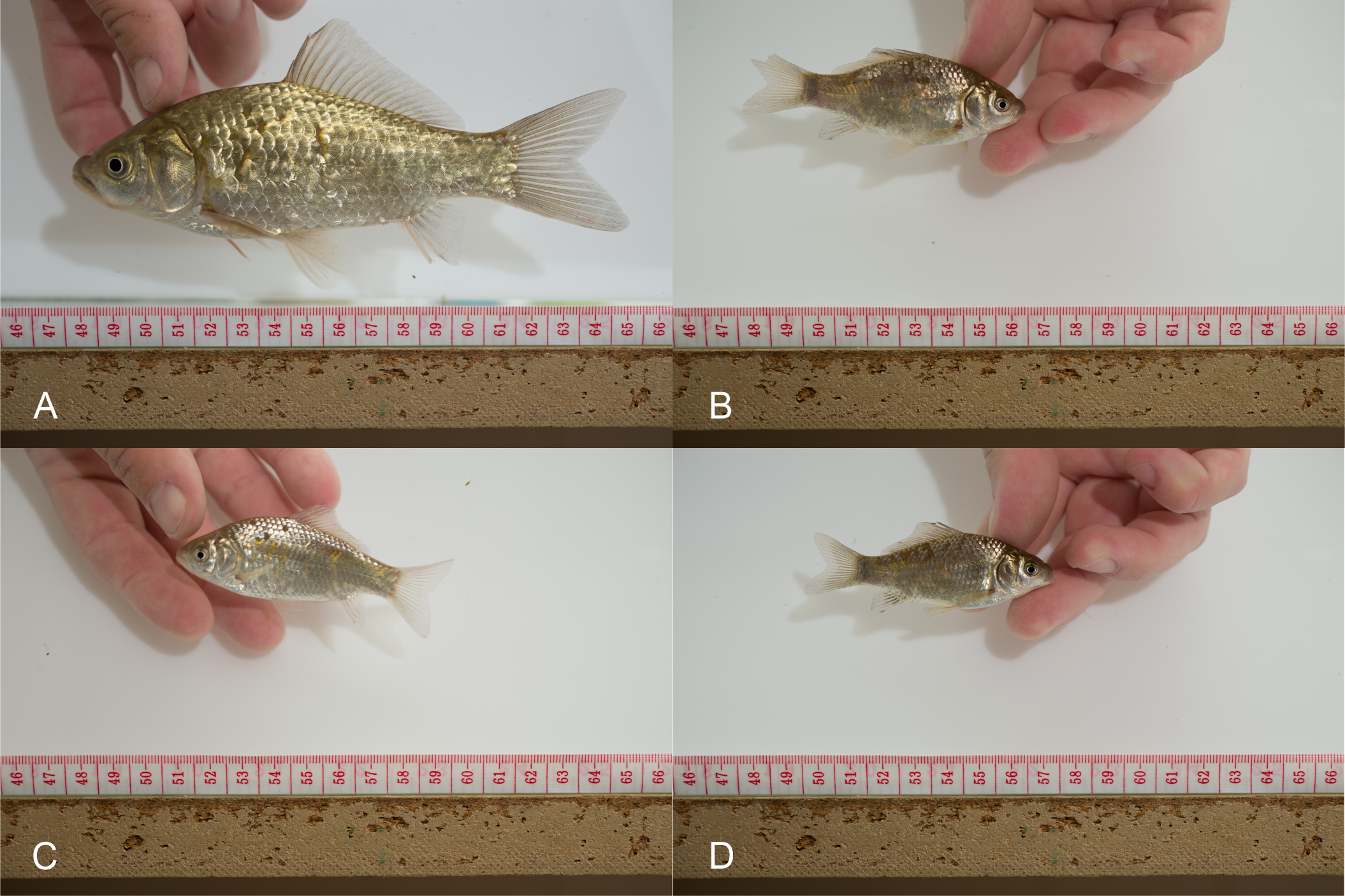

Supplement: S1 Fig — A) C. langsdorfii, B-D) C. gibelio. (TIF) [file pone.0268694.s001.tif]
