# Supplementary material for: A comprehensive DNA barcode inventory of Austria’s fish species
Source: PLoS One. 2022 Jun 9;17(6):e0268694. doi: 10.1371/journal.pone.0268694 (PMC9182252; doi:10.1371/journal.pone.0268694)
Supplement: S1 Table — (DOCX) [file pone.0268694.s002.docx]

Table S1: Summary of species delimitation analyses results.

|  | | **Group No.** | | |
| --- | --- | --- | --- | --- |
| **Species** | **BIN** | **ABGD** | **ASAP** | **bPTP** |
| *Acipenser ruthenus* | BOLD:AAA8921 | 73 | 76 | 46 |
| *Acipenser stellatus* | BOLD:AAA3851 | 80 | 83 | 45 |
| *Huso huso* | BOLD:AAA3852 | 79 | 82 | 44 |
| *Lepomis gibbosus* | BOLD:AAA5641 | 11 | 11 | 1 |
| *Rhodeus amarus* | BOLD:AAC4093 | 40 | 40 | 10 |
| *Cobitis elongatoides* | BOLD:ACE4983 | 61 | 63 | 15 |
| *Misgurnus bipartitus* | BOLD:ACB5380 | 8 | 8 | 14 |
| *Misgurnus fossilis* | BOLD:AAK6219 | 42 | 42 | 8 |
| *Sabanejewia balcanica* | BOLD:AAE3193 | 22 | 22 | 7 |
| *Barbus balcanicus* | BOLD:AAC5468 | 75 | 78 | 57 |
| *Barbus barbus* | BOLD:AAD1959 | 25 | 25 | 56 |
| *Carassius auratus* | BOLD:AAA7176 | 64 | 66 | 61 |
| *Carassius carassius* | BOLD:AAN9565 | 72 | 74 | 54 |
| *Carassius gibelio* | BOLD:AAA7176 | 64 | 66 | 61 |
| *Carasssius langsdorfii* | BOLD:AAA7176 | 81 | 84 | 62 |
| *Cyprinus carpio* | BOLD:AAA7175 | 34 | 34 | 25 |
| *Gobio* spp. | BOLD:AAC5607; BOLD:ADH1249; BOLD:ABY6890 | 55, 56, 59 | 57, 58, 61 | 87, 88 |
| *Pseudorasbora parva* | BOLD:AAD0138 | 50 | 51 | 9 |
| *Romanogobio carpathorossicus* | BOLD:ABV4495 | 51 | 52 | 30 |
| *Romanogobio skywalkeri* | BOLD:ADH6027 | 58 | 60 | 31 |
| *Romanogobio uranoscopus* | BOLD:AAF7823 | 52 | 53 | 60 |
| *Romanogobio vladykovi* | BOLD:AAC5609 | 36 | 36 | 59 |
| *Abramis brama* | BOLD:AAC8592 | 5 | 5 | 51 |
| *Alburnoides bipunctatus* | BOLD:AAC4344 | 63 | 65 | 26 |
| *Alburnus alburnus* | BOLD:AAB6906 | 26 | 26 | 71 |
| *Alburnus chalcoides* | BOLD:AAB6908 | 48 | 49 | 72 |
| *Ballerus ballerus* | BOLD:AAZ6088 | 87 | 90 | 79 |
| *Ballerus sapa* | BOLD:AAF3389 | 46 | 47 | 78 |
| *Blicca bjoerkna* | BOLD:AAD3588 | 83 | 86 | 68 |
| *Chondrostoma nasus* | BOLD:AAD7920 | 6 | 6 | 38 |
| *Leucaspius delineatus* | BOLD:ACF4430 | 85 | 88 | 35 |
| *Leuciscus aspius* | BOLD:AAC8137 | 29 | 29 | 52 |
| *Leuciscus idus* | BOLD:AAD5733 | 1 | 1 |  |
| *Leuciscus leuciscus* | BOLD:AAD5733 | 1 | 1 | 53 |
| *Pelecus cultratus* | BOLD:AAF5575 | 86 | 89 | 16 |
| *Phoxinus lumaireul* | BOLD:AAC8034 | 4, 7, 78 | 4, 7, 81 | 80, 82, 83 |
| *Phoxinus phoxinus* | BOLD:AAC8034;  BOLD:AAC8036; BOLD:ADL2661; BOLD:ACE5740 | 14, 15, 41 | 14, 15, 41, 55 | 49, 50, 81, 84 |
| *Rutlius meidingeri* | BOLD:AAA5494 | 18 | 18 | 55 |
| *Rutilus rutilus* | BOLD:ABZ3785 | 27 | 27 | 58 |
| *Rutilus virgo* | BOLD:AAE3231; BOLD:ADG8651 | 62, 84 | 64, 87 | 76, 77 |
| *Scardinius erythrophthalmus* | BOLD:AAC1452 | 45, 89 | 45, 46 | 34 |
| *Squalius cephalus* | BOLD:AAD8346 | 44 | 44 | 40 |
| *Telestes souffia* | BOLD:AAE9853 | 54 | 56 | 39 |
| *Vimba vimba* | BOLD:AAD9149 | 37 | 37 | 67 |
| *Barbatula barbatula* | BOLD:AAA1239; BOLD:AAA1243 | 57, 65 | 59, 67 | 47, 48 |
| *Tinca tinca* | BOLD:AEJ6454 | 69, 90 | 71, 75 | 22 |
| *Ctenopharyngodon Idella* | BOLD:ACL1923 | 32 | 32 | 29 |
| *Hypophthalmichthys molitrix* | BOLD:AAF6633 | 38 | 38 | 65 |
| *Hypophthalmichthys nobilis* | BOLD:ADK6840 | 88 | 91 | 66 |
| *Esox Lucius* | BOLD:AAA5988 | 33 | 33 | 4 |
| *Umbra krameri* | BOLD:AAO6269 | 71 | 73 | 5 |
| *Lota lota* | BOLD:AAB2046 | 19 | 19 | 3 |
| *Babka gymnotrachelus* | BOLD:AAX5968 | 82 | 85 | 24 |
| *Neogobius melanostomus* | BOLD:AAC0218 | 13, 74 | 13, 77 | 73, 74, 75 |
| *Ponticola kesslerii* | BOLD:AAD8740 | 28 | 28 | 23 |
| *Proterorhinus semilunaris* | BOLD:AAD0669 | 21 | 21 | 17 |
| *Cottus gobio* | BOLD:ABX6144 | 16 | 16 | 2 |
| *Gasterosteus aculeatus* | BOLD:AAA8488 | 49 | 50 | 12 |
| *Pungitius pungitius* | BOLD:AAA8317 | 10 | 10 | 11 |
| *Gymnocephalus baloni* | BOLD:AAL5632 | 76 | 79 | 42 |
| *Gymnocephalus cernua* | BOLD:ACO0744 | 66 | 68 | 41 |
| *Gymnocepahlus schraetser* | BOLD:AAB0394 | 39 | 39 | 43 |
| *Perca fluviatilis* | BOLD:AAB0356 | 9 | 9 | 18 |
| *Sander lucioperca* | BOLD:AAD1749 | 30 | 30 | 69 |
| *Sander volgensis* | BOLD:AAJ5463 | 77 | 80 | 70 |
| *Zingel streber* | BOLD:AAE6523 | 31 | 31 | 37 |
| *Zingel zingel* | BOLD:AAH8409 | 12 | 12 | 36 |
| *Eudontomyzon mariae* | BOLD:ABY5382 | 2 | 2 | 63 |
| *Lampetra planeri* | BOLD:AAB6058 | 3 | 3 | 64 |
| Coregonus spp. | BOLD:ACA5470 | 17 | 17 | 21 |
| *Hucho hucho* | BOLD:AAE1471 | 23 | 23 | 20 |
| *Oncorhynchus mykiss* | BOLD:AAA1627 | 53 | 54 | 19 |
| *Salmo salar* | BOLD:AAA3435 | 43 | 43 | 27 |
| *Salmo trutta* | BOLD:AAB3872 | 60 | 62 | 28 |
| *Salvelinus alpinus* | BOLD:ABZ0871 | 20 | 20 | 32 |
| *Salvelinus fontinalis* | BOLD:AAC3575 | 35 | 35 | 33 |
| *Salvelinus umbla* | BOLD:ABZ0871 | 20 | 20 | 32 |
| *Thymallus thymallus* | BOLD:AAD6463 | 24, 47 | 24, 48 | 13 |
| *Ameiurus melas* | BOLD:AAA7255 | 70 | 72 | 86 |
| *Ameiurus nebulosus* | BOLD:AAA7255 | 67 | 69 | 85 |
| *Silurus glanis* | BOLD:ACL1933 | 68 | 70 | 6 |
