# Supplementary material for: A comprehensive DNA barcode inventory of Austria’s fish species
Source: PLoS One. 2022 Jun 9;17(6):e0268694. doi: 10.1371/journal.pone.0268694 (PMC9182252; doi:10.1371/journal.pone.0268694)
Supplement: Attachment — Submitted filename: Response_to_Reviewers.docx [file pone.0268694.s003.docx]

Response to Reviewers

Journal requirements:

1. Please ensure that your manuscript meets PLOS ONE's style requirements, including those for file naming. The PLOS ONE style templates can be found at

https://journals.plos.org/plosone/s/file?id=wjVg/PLOSOne_formatting_sample_main_body.pdf and

<https://journals.plos.org/plosone/s/file?id=ba62/PLOSOne_formatting_sample_title_authors_affiliations.pdf>

**Response:** Done.

2. We note that the grant information you provided in the ‘Funding Information’ and ‘Financial Disclosure’ sections do not match.

When you resubmit, please ensure that you provide the correct grant numbers for the awards you received for your study in the ‘Funding Information’ section.

**Response:** Done.

3. We note that you have stated that you will provide repository information for your data at acceptance. Should your manuscript be accepted for publication, we will hold it until you provide the relevant accession numbers or DOIs necessary to access your data. If you wish to make changes to your Data Availability statement, please describe these changes in your cover letter and we will update your Data Availability statement to reflect the information you provide.

**Response:** We provided Genbank Accession Numbers and DOIs of datasets in the revised version.

4. Please include a copy of Table 1 which you refer to in your text on page 9.

**Response:** Done. Table 1 was included in the manuscript.

5. Please include captions for your Supporting Information files at the end of your manuscript, and update any in-text citations to match accordingly. Please see our Supporting Information guidelines for more information: <http://journals.plos.org/plosone/s/supporting-information>.

**Response:** Done. We have included all figures and table captions as well as the captions for the supporting material.

6. Please review your reference list to ensure that it is complete and correct. If you have cited papers that have been retracted, please include the rationale for doing so in the manuscript text, or remove these references and replace them with relevant current references. Any changes to the reference list should be mentioned in the rebuttal letter that accompanies your revised manuscript. If you need to cite a retracted article, indicate the article’s retracted status in the References list and also include a citation and full reference for the retraction notice.

**Response:** Done.

Reviewer #1

Abstract: line 32: should also Gobio sp be referred here?

**Response:** Yes, we included “*Gobio* spp.” here in the revised version as well.

48: “determine different ontogenetic stages”: this phrase is not clear. DNA barcoding cannot determine different ontogenetic stages but can identify species regardless of the ontogenetic stage. Please rephrase.

**Response:** We changed it to “…determine species regardless of their ontogenetic stage…”

Materials and methods:

Authors firstly present the dataset they used: how these sequences were produced? You should rearrange the materials and methods starting from the collection of the samples

154-155: in “materials and methods” section authors refer that 681 barcodes whereas here this number is 740. Which is the correct one? Additionally, how the overall success rate of 93% is calculated?

**Response:** The reviewer is correct in his statement, that we have switched the order in which newly generated and already publicly available downloaded sequences are presented in the M&M section. However, in our opinion, this doesn’t impede readability or understandability and thus we kept this particular order. Nonetheless, we rephrased and rearranged this section slightly: It now states the two sources of data in the beginning, then explains the rationale behind excluding the majority of publicly available sequences, then describes all procedures regarding the fresh material and then sums up the final dataset before going into details about the different analyses.

Furthermore, we corrected and unitized the number of samples and respective barcodes throughout the entire manuscript. The overall success rate represents the ratio of successfully sequenced samples compared to the overall number of samples. We added ‘sequencing success rate’ in order to increase understandability for the reader.

164-173: This part is a bit confusing, especially the last sentence. In the first sentence you are referred to multiple BINS for one species (or genus), then to BIN sharing and in the last one to multiple BINS for one species (again). I assume that as regards multiple BINS in the first case you mean the BINS which are produced by your results whereas in the second case one BIN comes from the present study and the second one from BOLD data (considering also the Table S1). Please explain better what you mean.

**Response:** Initially, we separated those results because in the first three cases the different BINs likely correspond to different species, whereas in the latter they just indicate intraspecific variation. However, we rearranged this paragraph and now state all cases of multiple BINs for one species/genus and then cases of BIN sharing. This should improve clarity and readability and the individual cases are in any case discussed later in the manuscript.

199: “North Macedonia” instead of “Makedonia”

**Response:** We changed it accordingly.

215: why missing? Authors may explain why these species are missing

**Response:** We added the sentence: “For the two former species, PCRs (of old museum tissue) were unsuccessful, for the latter species no samples could be obtained” as an explanation.

215-219: Would 1-2 individuals be too little to represent a species? Can we ensure the accuracy of the morphological identification on-site?

**Response:** A single or two DNA barcodes could be considered sufficient to represent a species, especially when the specimen from which a barcode was obtained, can be identified morphologically with certainty. Furthermore, the *COI* sequences of those species are distinctly different to the sequences of their most closely related sister species thus excluding the possibility of misidentification. However, in terms of genetic and geographic diversity of course, 1-2 specimens are insufficient and do not allow for the estimation of intraspecific variation. Nonetheless, for identification purposes, a single high-quality sequence of an unambiguously determined reference specimen should be enough, given that intraspecific variation is well exceeded by interspecific genetic distances (i.e., the ‘Barcode gap’ exists).

Finally, two more general comments are presented:

1. It would be useful to present the number of individuals per species (only for a few species it is clear in the manuscript) (eg in a table).

**Response:** We agree with the reviewer and like to direct attention to Table 1, where a column ‘N’ already exists, stating the number of sequences per species. Also, we included a new link to Table 1 in the M&M section.

2. Authors may include a map including all the collection sites.

**Response:** Done. We have included a sampling map as a new figure 1 and update all subsequent figure numbers.

3. Please provide Genbank accession numbers and BOLD IDs for the sequences produced by the present study (I assume that you have already submitted them).

**Response:** We included the Accession Numbers and DOIs for all datasets in the manuscript.

Reviewer #2

The introduction is most focused on the method of Barcode and how this tool is effective to studies in general. However, I missed more information about Austria fish fauna, the last assessment, and the biodiversity known. So, the reader will be able to compare with the results of the present study.

I also did not see any legend for the figures (except by fig.2), as well as for the tables.

**Response:** The reviewer might have missed it, but we did (and still do) dedicate an entire paragraph to the situation of fish in Austria, including information on Red List assessment, species number, counts of native and alien species as well as new additions to the present fish fauna.

We added the information when the last Red List assessment was conducted, but in the first sentence of the last paragraph of the introduction section.

Furthermore, we included all figure and table captions in the new version of the manuscript.

Title: remove “lampreys” from the title since they are also fish. I do not understand why authors mentioned as a separate group. So, change the title to “A comprehensive DNA barcode inventory of Austria’s fish species”

**Response:** The distinction between fish (Osteichthyes) and lampreys (Cyclostomata) derives from German colloquial language. Although, this distinction has also been used in other international scientific articles (e.g., Knebelsberger, T., Dunz, A. R., Neumann, D., & Geiger, M. F. (2015). Molecular diversity of Germany's freshwater fishes and lampreys assessed by DNA barcoding. Molecular ecology resources, 15(3), 562-572.), we follow the reviewer’s suggestion and use “fish species” throughout this manuscript. However, we have written out the distinction between teleost fish and lampreys in the introduction in order to convey this content to the reader and increase visibility for researchers looking for either or both groups.

Line 20-21: once more why lampreys are treated as separate from fish since it is also fish?

**Response:** See response above for explanation. We deleted “lampreys” here.

Line 21: change “Red List” for “Red List of threatened species”

**Response:** We changed it accordingly.

Line 22: delete “lamprey species”

**Response:** Done.

Line 24: change “inventory” for “database”; delete “lamprey species”

**Response:** We changed it accordingly.

Line 25: change “COI” for “coI”. There is an agreement to standardize the abbreviation of gene names, and particularly for fish, in which all codified genes for fish groups should be abbreviated in lower case and italic.

**Response:** We did not follow the reviewer’s suggestion to change *COI* for *coI*. The way gene abbreviations are written depends on the journal and not the study taxa. As other publications in PLoS One use “*COI*” as well (Mitchell, A., & Gopurenko, D. (2016). DNA barcoding the Heliothinae (Lepidoptera: Noctuidae) of Australia and utility of DNA barcodes for pest identification in Helicoverpa and relatives. *PLoS One*, *11*(8), e0160895.; Tsoupas, A., Papavasileiou, S., Minoudi, S., Gkagkavouzis, K., Petriki, O., Bobori, D., ... & Triantafyllidis, A. (2022). DNA barcoding identification of Greek freshwater fishes. *PloS one*, *17*(1), e0263118.; Foottit, R. G., Maw, E., & Hebert, P. D. N. (2014). DNA barcodes for nearctic auchenorrhyncha (Insecta: Hemiptera). *PloS one*, *9*(7), e101385.; Kochzius, M., Seidel, C., Antoniou, A., Botla, S. K., Campo, D., Cariani, A., ... & Blohm, D. (2010). Identifying fishes through DNA barcodes and microarrays. *PloS one*, *5*(9), e12620.), we decided not to use “*coI*”.

Line 27: delete “almost perfectly”

**Response:** Here, we kindly disagree with the reviewer and keep the present wording. In our opinion, “almost perfectly” is suitable here as the number of genetic clusters almost exactly matches the number of (morphologically) currently accepted species.

Line 28: what do you mean in this case with morphospecies? Since the analysis resulted in undescribed species, this new morphotypes were already known to science? I think only “species” is more suitable here and then you can add new forms for the probably new species or distinct lineages.

**Response:** We agree with the reviewer and changed it to species.

Line 48: I am not sure if this is possible, as I did not read the paper mentioned. But, I think maybe you want to mean “identify juveniles and larvae rather than determine different ontogenetic stages. The molecular is equal for adults or juveniles, so I did not get it. Please revise this.

**Response:** We changed it to “…determine species regardless of their ontogenetic stage…”

Line 106: change for “coI sequences of Austrian fish species already available from BOLD…”

**Response:** Done.

Line 107: space between 681 and new

**Response:** Done.

Line 108-112: You do not need inform the total number of sequences before you remove the unidentified species or shorter ones. Present the number used in the analysis (i.e. 1,014) and remain the observation that you excluded the unidentified species and shorter sequences from the analysis. The number of thousand should be present as 1,014.

**Response:** We agree with the reviewer that it would not be necessary to include the full number of available sequences here. However, we wanted to provide this information due to the higher number of excluded records and in order to make it reproducible based on our rationale.

Line 113: change “non-Austrian sequences from elsewhere in Europe” for “sequences from other region of Europe, outside of Austria, were used from BOLD…”

**Response:** We changed it accordingly.

Line 119: which Museum? Different museum ? listed on…?

**Response:** We added the names of the museums that contributed samples in the text. The information where the samples are stored is given in the next sentence directing the reader to the BOLD project including all sampling and storage information.

125: which were the parameters for the amplification of coI? It should be written here.

**Response:** Since we have referenced the wet lab protocol for PCR amplification and cycle sequencing as well as the clean-up, we opted not to go into further details concerning the PCR conditions etc. here. All necessary information to reproduce our procedures can be extracted from those references. However, we included the polymerase we used for PCRs as well as the annealing temperature.

Line 154: How could be 740 barcode sequences if you previously mentioned 742?

**Response:** Here, as well as in the M&M section we distinguish between two different counts. One indicates the total number of samples, the other one only takes into account the successfully sequenced samples (i.e. the number of newly generated DNA barcode sequences). The ratio of these two metrics is presented as the overall sequencing success rate of 93%. We checked, corrected and unified the number of samples and newly generated DNA barcodes throughout the manuscript.

Line 155: overall success rate of what? Of identification at species level? Something is missing here. I also think that present the covering data in number not in percentage is more reliable since we can have a better ideia about it. Or, as the covering is high perhaps mention how species the study did not contemplate.

**Response:** We meant the sequencing success rate, meaning the ratio of successfully sequenced samples compared to the overall number of collected samples. We inserted ‘sequencing success rate’ in order to increase understandability. However, we state both absolute numbers as well as the rate in percent in this very sentence thus we would leave the rest of the sentence as it currently is.

Line 158; there isn`t “well resolved” in phylogenies and tree. It is resolved or not. Perhaps you meant “well supported”. My suggestion is delete “well” from the sentence.

**Response:** We replaced “well resolved” by “distinct”.

Line159: Also, there is not “perfectly” in terms of taxonomy, so delete this word from the text, and change for “the clades match with morphological species identification”.

**Response:** We deleted “perfectly” according to the reviewer’s suggestion and changed the phrase to “largely mirroring”, since the number of currently accepted morphologically recognized species slightly deviates from the number of clusters on the NJ tree as well as BINs recovered.

Line 163: this example of C. langsdorfii is was not a discordance between DNA and morphospecies, actually this is not the conceptual of morphospecies. This was an clear example of a misidentification.

**Response:** We agree with the reviewer that this was a case of misidentification. We changed the following sentence (see response below) to avoid misleading terminology. Furthermore, we changed the beginning of the following sentence to ‘In addition to…’ in order to emphasize the enumeration of individual/separate insights derived from the NJ tree rather than to make it sound like the finding of *C. langsdorfii* is one of the discordances between accepted species and genetic identification.

Line 164: change morphospecies for morphotypes. Morphospecies is other conceptual and need very different analysis to show the space of the shape of the species. Not about different phenotypes.

**Response:** We agree with the reviewer and changed it to ‘currently accepted species’.

Line 177: This could be also a case of molecular contamination sample during Lab techniques.

**Response:** We ruled out this possibility due to reproduction of the result after re-extraction of DNA from the tissue as well as re-amplification and re-sequencing.

Line 177 and 178: What do you mean with Lmax? It is not mentioned in M&M

**Response:** Imax means maximum intraspecific distance and is explained in the caption of Table 1. Furthermore, we included it in the M&M section as well as in the Results section.

Line 180-183: sort species in alphabetic order.

**Response:** Done, we sorted the species alphabetically.

Line 191: cite for the first time the entire name of the genus

**Response:** Done. We have written out the full name of the genus.

Line 315 and 316: There is not “true” for the species. Change for stricto sensu

**Response:** We changed it accordingly.

Line 323: What do you mean with “physical” museum collection?

**Response:** The term ‘physical museum collection’ was meant to distinguish between physical specimens compared to digital repositories (of e.g. DNA sequences, like Genbank or BOLD) storing information connected to physical specimens. However, we have deleted “physical” in the revised version of the manuscript.

Line 324 and 325: The phrase is understandable.

**Response:** We have deleted “The dynamics of labelling and subsequent re-evaluations”.

Line 325-326: This is not an example of a issue, since the species is the same, it is matter of with genus is included the species. Doesn’t change the ID of the species.

**Response:** Here we disagree with the reviewer. It is not a matter of which genus applies, as *Romanogobio* is the currently accepted genus. We have slightly altered the text to avoid this confusion. In the next sentence, we provide the information that the species epithet itself has changed (not just the genus) based on the information provided by Friedrich et al. (Friedrich, T., Wiesner, C., Zangl, L., Daill, D., Freyhof, J., & Koblmueller, S. (2018). *Romanogobio skywalkeri*, a new gudgeon (Teleostei: Gobionidae) from the upper Mur River, Austria. Zootaxa, 4403(2), 336-350.)). In fact, *Romanogobio kesslerii* constitutes an entirely different species with its own distribution area, which is not connected to *R. carpathorossicus*. However, in the recent past, specimens of *R. carpahtorossicus* have been identified as *R. kesslerii* in Austria, thus hypothetically extending the alleged distribution range of *R. kesslerii* substantially. This also led to erroneously determined/labelled specimens being deposited in museum collections as well as DNA sequences being uploaded under a ‘false flag’. Potential repercussions of such labelling issues could affect entire cascades of data manipulation and interpretation leading for example to flawed threat and Red List assessments.

Line 328: This is not about to decide between the use of the name R. kessleri (or G. kessleri) or R. carpathorossicus. It is about if R. carpathorossicus is a valid species or not. If not (in case of junior synonym of R. kessleri), thus the it should use the prevalence rule of the oldest name, i.e. R. kessleri.

**Response:** According to Friedrich et al. (Friedrich, T., Wiesner, C., Zangl, L., Daill, D., Freyhof, J., & Koblmueller, S. (2018). *Romanogobio skywalkeri*, a new gudgeon (Teleostei: Gobionidae) from the upper Mur River, Austria. Zootaxa, 4403(2), 336-350.)), *R. kesslerii* is an entirely different species than *R. carpathorossicus* and for specimens found in Austria (which have been determined as *R. kesslerii* in the recent past), in our view *R. carpathorossicus* is the correct species name. See also our response to the comment above.

Line 339: the repositories is not for reflect the current accept nomenclature but rather for support the nomenclature decisions.

**Response:** Yes, such/all repositories can support nomenclatural decisions due to the material/information they house. However, incorrect/inadequate/outdated nomenclature may well pose a source for errors, especially, when used by people who are not expertly familiar with a certain group or taxon. For example, lending samples from a museum collection under outdated labels for genetic/genomic analyses may lead to false uploads to sequence repositories and erroneous deductions from the generated results (Mulcahy, D. G., Ibáñez, R., Jaramillo, C. A., Crawford, A. J., Ray, J. M., Gotte, S. W., ... & de Queiroz, K. (2022). DNA barcoding of the National Museum of Natural History reptile tissue holdings raises concerns about the use of natural history collections and the responsibilities of scientists in the molecular age. *Plos one*, *17*(3), e0264930.). Furthermore, at least in our opinion, it would be rather impractical to have to look up outdated nomenclature to find the currently accepted name in order to be able to work on a particular taxon.

Figures: except by Fig. 2, they don`t have legend, as well as the supplementary tables.

**Response:** We included all figure, table and supplementary materials captions in the revised version.
